# Accidental Trisodium Phosphate [Na_3_ PO_4_] (TSP) Ingestion in a Child

**DOI:** 10.1111/jpc.70012

**Published:** 2025-02-12

**Authors:** William Hoffmann, Christopher Cooke, Ryan F. Bloomquist

**Affiliations:** ^1^ School of Medicine University of South Carolina Columbia South Carolina USA

**Keywords:** caustic ingestion, trisodium phosphate, TSP

## Abstract

**Background:**

It is not uncommon for a child to present to the hospital due to accidental ingestions, and oftentimes they are the result of a child accessing household cleaning or home improvement products. Typically, the upper gastrointestinal tract is the site of initial tissue insult and prognosis of these cases depends on a variety of factors, including the ingested substance, injury extent, and assessments and treatments rendered. Possible post‐ingestion complications that make management difficult may include mediastinitis, hemodynamic instability, gastrointestinal perforation, erosion and scarring.

**Case:**

In this case, a 13‐month‐old boy accidently swallowed Trisodium phosphate [Na_3_PO_4_] (TSP), a common household all‐purpose heavy‐duty cleaner. After ingestion, the patient presented to the emergency department where a multi‐disciplinary team‐initiated care. Work‐up included regular vital checks, electrolyte profiles, blood profiles and an esophagogastroduodenoscopy. He was eventually discharged and scheduled to return for follow‐up with esophagram, without long term consequences.

**Conclusions:**

Although the ingestion of TSP has occurred before, the literature regarding consumption of this specific detergent is negligible. This case provides evidence regarding the treatment and outcome of a paediatric patient who accidentally swallowed TSP and offers guidance in the management of their care.

## 1 Introduction

The leading cause of death for children of the ages 1–9 years old are accidents (unintentional injuries) [1] and one of those unintentional injury categories is caustic ingestion. Caustic ingestion cases are more likely to occur in children less than 5 years old, with a peak incidence at the age of 2 years old [2]. The most common ingested products include household or home improvement chemicals, such as bleach, detergents and industrial paint products. Here, the severity of injuries depends on several factors and when assessing the agent in a caustic ingestion case, the pH, concentration, volume and the amount of time that has elapsed since ingestion need to be considered [3]. Agents at the different extremes of the pH scale are very destructive to the mucosal surfaces that line the gastrointestinal tract. Alkaline agents when ingested can lead to liquefactive necrosis, which disrupts cell membranes and can cause cells to lyse, releasing degradative proteins damaging nearby tissue [4]. Acids tend to result in coagulative necrosis and this coagulum limits spread and penetration into surrounding tissues [5]. For this reason, swallowing alkaline agents generally carry higher morbidity. Moreover, solid agents are more likely to damage the upper oesophagus to oral pharynx due to adherence to the mucosa, whereas liquid agents are typically more readily dispersed. In the gastrointestinal tract, the oesophagus and stomach tend to be the organs most impacted by caustic ingestion while the small bowel is usually spared from injury due to reflexive pyloric spasms, which help mitigate the passage of toxins [6].

Trisodium phosphate [Na_3_PO_4_] (TSP) is an all‐purpose heavy‐duty cleaner, and in solution TSP has a pH of 12–14, making it highly caustic. This high pH affords utility in a variety of cleaning and degreasing scenarios, and it is commonly used as an effective cleaning agent to saponify grease and oils [7]. To date, there is little to no direct medical literature on the treatment and outcomes of patients who have accidentally ingested TSP. We present this case on the diagnostic work up and clinical management of TSP ingestion in a 13 month‐old male.

## 2 Case

**FIGURE 1:**
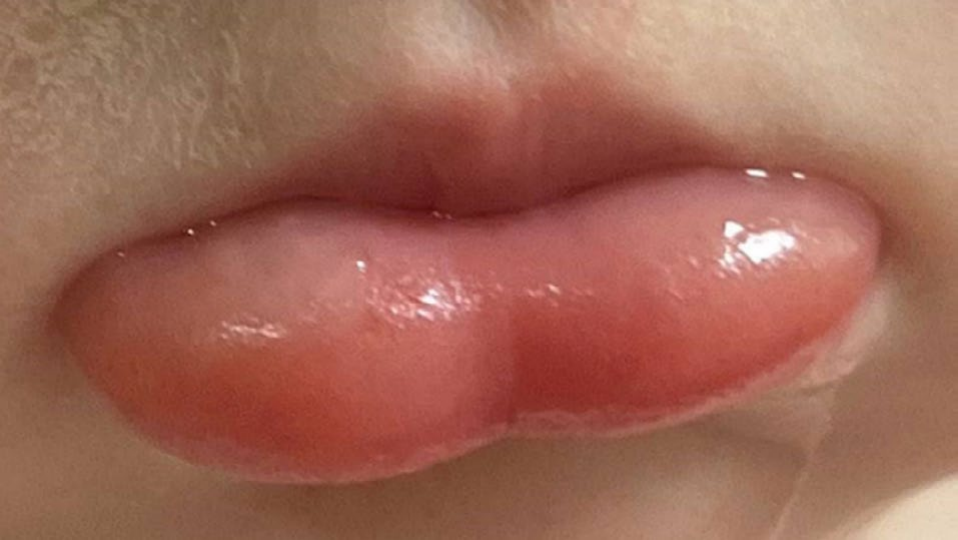
Diffuse lower lip swelling observed shortly after ingestion.

A previously healthy 13‐month‐old male, with no significant medical history presented to the emergency department about 2 hours after ingestion of a home cleaning product. His parents called emergency medical services (EMS) after discovering their child accidentally ingested a mouthful of a white powder. His parents suspected the white powder to be trisodium phosphate after finding him next to an open box of trisodium phosphate in the garage. The parents immediately attempted to wash out the child's mouth with water. Once EMS arrived to the scene, EMS reported the patient had significant lower lip swelling and noted that the parents informed them of two episodes of vomiting prior to their arrival (Figure 1).

**FIGURE 2:**
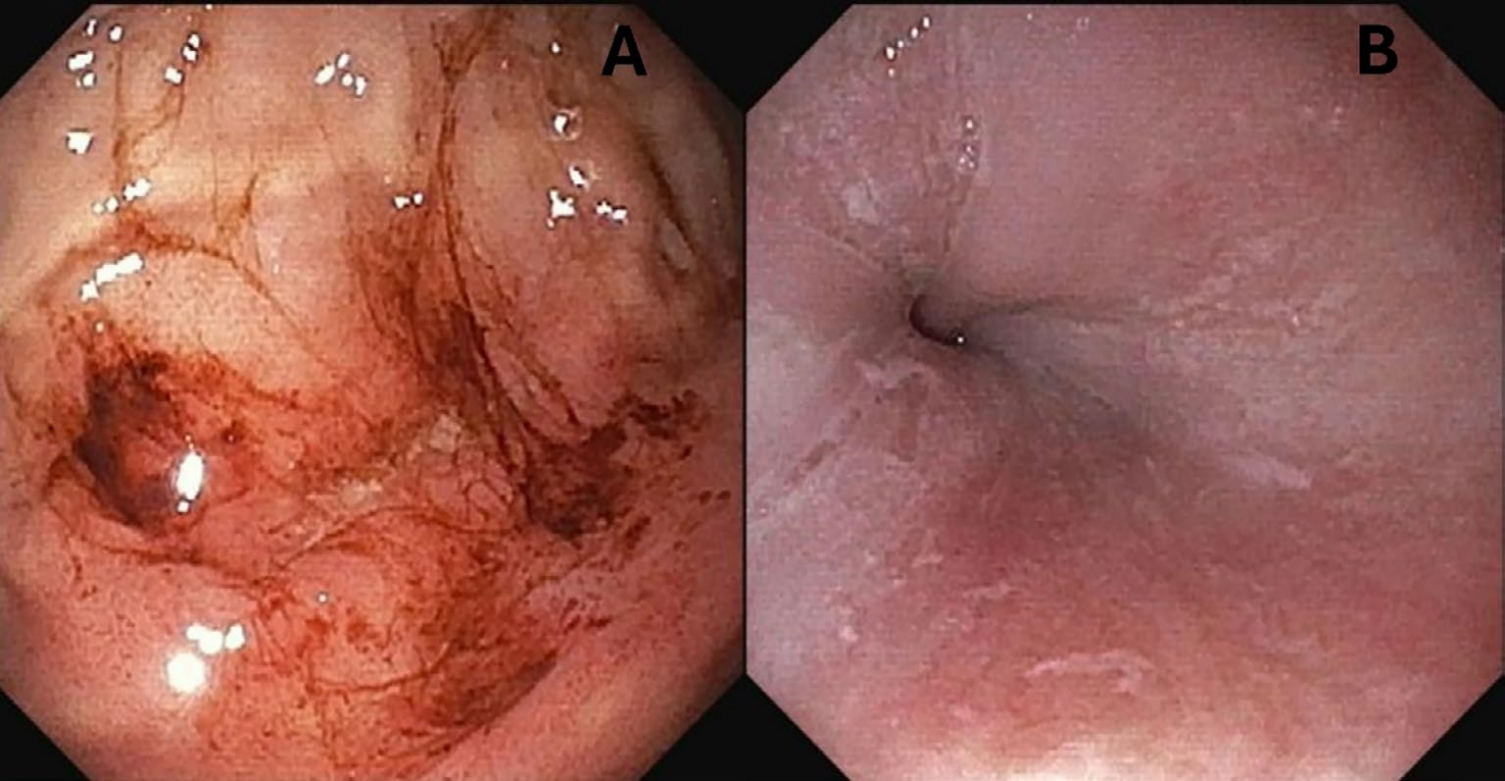
Image (A) shows scattered mild inflammation, and linear erosions were noted throughout the stomach. Image (B) shows diffuse moderate mucosal change and white specks found throughout the oesophagus.

On route to the emergency department, poison control was contacted. Their recommendations included a comprehensive assessment with a complete blood count and electrolyte evaluation. Additionally, they advised a period of observation spanning at least 4–6 hours, alongside gastrointestinal (GI) consultation for potential endoscopic examination due to the delayed risk of oesophageal caustic injury. His vitals were stable with a heart rate (HR) of 127 bpm, blood pressure (BP) of 96/54 mm Hg (mmHg), an oral temperature of 98.1°F, respiratory rate (RR) of 30 respirations/min, and a peripheral oxygen saturation (SpO_2_) of 98% measured with a finger pulse oximeter. Normal vitals for a toddler (1–2 years old) consist of a HR of 98–140 bpm, a BP of (86–106)/(42–63) mmHg, an oral temperature of 97.9°F–100.4°F (36.6°C–38°C), a RR of 22–37 respirations/min and a SpO_2_ of 95%–100% [8]. The physical exam in the emergency department revealed lower lip swelling, tongue desquamation and posterior pharyngeal erythema. Physical examination did not reveal any respiratory signs of struggle or distress, such as stridor, wheezing or dyspnea. With no signs of respiratory distress, laryngoscopy was not pursued. Initial laboratory investigations were largely unremarkable, only revealing a minor metabolic acidosis characterised by a bicarbonate level of 19 and a mild transaminitis. Considering the potential for co‐ingestion, assessments for acetaminophen and salicylate levels were conducted, all of which returned within normal limits. The patient was admitted to the Paediatric Intensive Care Unit (PICU) for stabilisation and observation given his unclear clinical status. While in the ICU, the patient's respiratory status remained stable with the patient not requiring intubation or other respiratory support. Subsequent monitoring revealed a declining trend in bicarbonate levels over the next 2 days, reaching a low of 13 without a concomitant elevation in anion gap. Notably, electrolyte profiles remained within normal ranges, except for a proportional rise in chloride levels. His vitals remained stable throughout his ICU stay. Following stabilisation after the initial day in the ICU, the patient was transferred to a general ward. On Day 2 post‐ingestion, gastrointestinal specialists recommended an esophagogastroduodenoscopy (EGD), which was performed on Day 2 and showed revealed diffuse moderate mucosal changes characterised by inflammation throughout the oesophagus and stomach (Figure 2).

**FIGURE 3:**
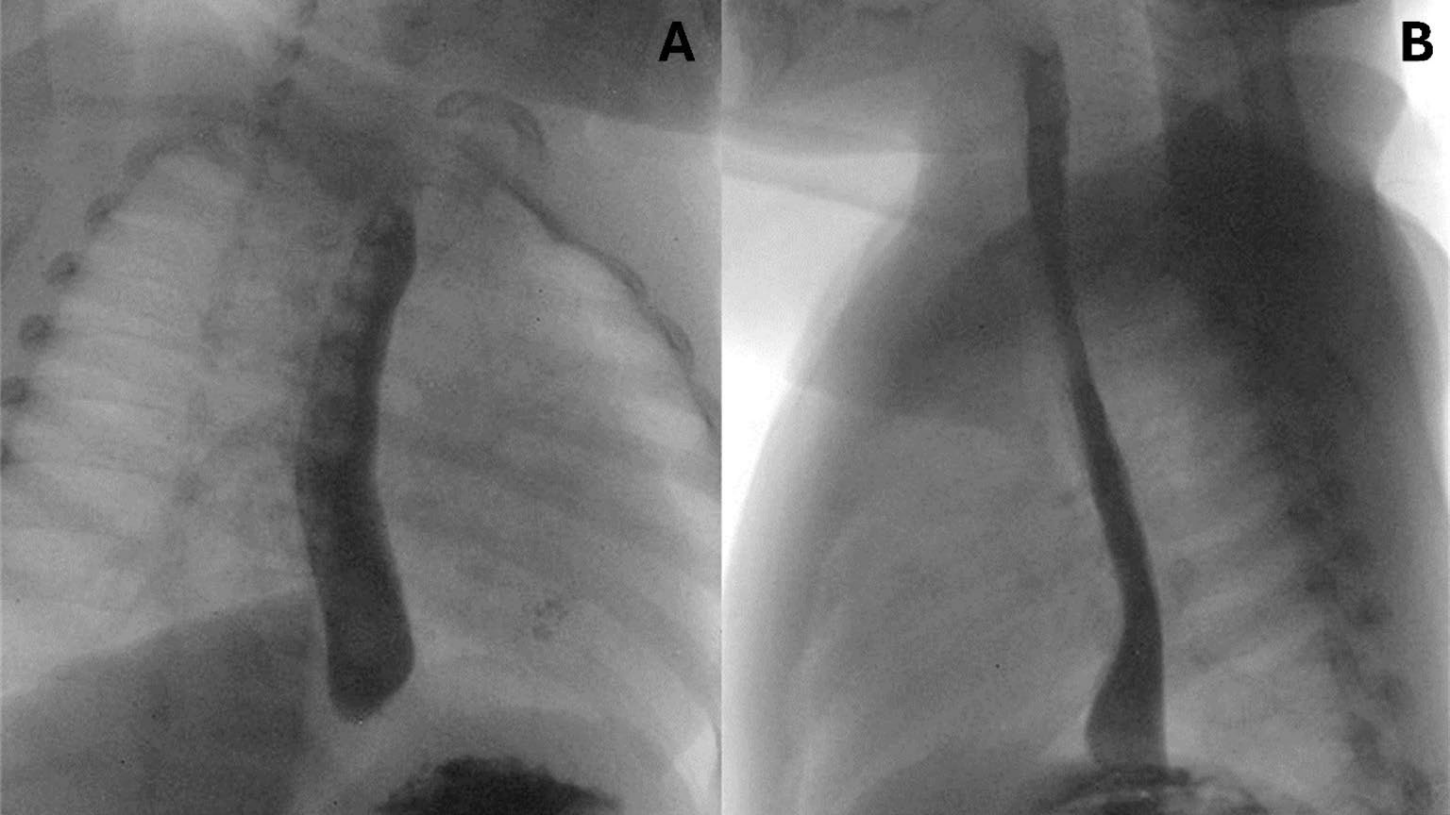
Esophogram performed at Day 2 post‐exposure. Image (A) (AP view) shows progression of contrast through oesophagus. Image (B) (Lateral view) shows progression of contrast into the stomach without evidence of leak.

There were longitudinal markings and white specks found in the entire oesophagus. Scattered mild inflammation was seen and characterised by linear erosions in the entire stomach. No gross lesions were noted in the duodenal bulb, the first portion of the duodenum, or the second portion of the duodenum. Overall, estimated blood loss was minimal. Given these findings, the patient was maintained on a nothing by mouth (NPO) diet order and scheduled for an esophagram, which showed no evidence of perforation or leak of contrast, moderate gastroesophageal reflex, and no evidence of stricture (Figure 3).

Subsequent observation for a 24‐hours period demonstrated good tolerance to oral intake, and his metabolic acidosis and transaminitis both resolved on repeat testing. The patient was alert and active, without signs of distress, so the decision was made to discharge him with strict return precautions. He was discharged with Lansoprazole 10 mg once daily and sucralfate 150 mg four times daily for his oesophageal irritation and stomach inflammation. 22 days following the ingestion, a follow‐up esophagram revealed no evidence of stricture or ulceration, with mild–moderate GER noted on exam. After this evaluation, PPI and sulcrafate were discontinued given the overall lack of chronic oesophageal and stomach injury. The patient was noted to be back to his normal baseline at this check‐up, without evidence of scarring or long‐term manifestations of the incident. The patient's family was counselled on reasons for return to clinic, and 6 months later no issues from the incident were noted, suggesting that TSP was locally caustic, but tolerated well after proper management, without the need for further interventions.

## 3 Discussion

Ingestions of strong bases from cleaning products and other sources pose a significant risk of immediate tissue damage upon contact. These alkaline detergents lead to the destruction of soft tissues and mucous membranes in various anatomical areas, including the face, oropharyngeal strictures, airway and gastrointestinal tract. Unlike acids, which cause coagulation necrosis resulting in eschar formation that limits penetration, alkaline substances may lead to liquefaction necrosis. This type of necrosis facilitates deeper tissue penetration, increasing the likelihood of more significant transmural injury. Alkaline substances are typically colourless, relatively tasteless and viscous, contributing to slower transit through the oesophagus and consequently, additional risk for oesophageal injury.

The management of caustic injuries starts with securing the airway and stabilising the patient's condition. Since this patient was stable on presentation without signs of acute distress or airway involvement, early intubation was unnecessary. However, in cases like these, intubation may be required, ideally conducted with fiberoptic laryngoscopy to help visualise the epiglottis and larynx directly and to minimise the risk of further trauma. In cases of severe oropharyngeal edema, a surgical airway may be required. Esophagogastroduodenoscopy (EGD) is considered the gold standard for diagnosis of damage caused by alkaline ingestion, guiding subsequent management, and as a diagnostic test oesophageal for stricture at follow‐up [9, 10]. The general recommendation for endoscopy after caustic ingestion is to perform it within the first 48 h to minimise the risk of perforation during the healing phase. A follow‐up endoscopy is typically performed around the 3‐week mark to assess for more permanent oesophageal injuries. If there is no evidence of strictures or other chronic injuries at that time, a third esophagram is generally not necessary. While there is not a set specific guideline or algorithm for treating caustic injuries, many studies have utilise an endoscopic grading system called Zargars, an endoscopic classification scale, when trying to plan appropriate treatments for these patients. This system correlates each level of oesophageal injury at with a nine‐fold increase in morbidity and mortality and each grade can be used predictor of systemic complications and death, with patients of a grade III or higher at larger a larger risk [11]. Currently there are no guidelines on the role NG tube placement at the time of an EGD but in a study by Rathnaswami and Ashwin, they note NG tubes may be used as a means of removing any caustic material left in the GI tract [12]. With the gold standard of EGD, using an NG tube prior is no longer necessary because the insertion of a foreign object can lead to retching or vomiting increasing possible exposure to the ingested agent [13]. While an NG tube can assist with luminal integrity and be used as route for enteral feeding, placement of an NG tube should be discussed with the care team on an individual case basis. For example, some factors to consider would include extent of damage to upper GI tract structures, any signs of dysphagia or obstruction, coagulation status and goals of care. One of the bigger risks to placing an NG tube to consider with damaged upper GI structures is perforations [12]. While this patient did have a mild transaminitis, there was not any further work‐up performed to investigate this finding. There is not any research on the relation of TSP ingestion and transaminitis specifically. However, there are articles discussing the relationship between corrosive esophagitis and transaminitis. Although this patient did not end up having corrosive esophagitis from this caustic ingestion, case reports like this one in the future could be investigated more for a possible relationship [14]. To our knowledge, this is the first description of an ingestion of trisodium phosphate. This characterisation and case reports should aid in management and treatment of a common household chemical.

## Ethics Statement

This work was completed in compliance with CARE guidelines and the relevant organisational ethics oversight committee. All authors attest that they meet the current ICMJE criteria for Authorship.

## Consent

Patient consent for this work was provided by the patient guardian.

## Conflicts of Interest

The authors declare no conflicts of interest.
